# Comparison of the association of mammographic density and clinical factors with ductal carcinoma in situ versus invasive ductal breast cancer in Korean women

**DOI:** 10.1186/s12885-017-3841-0

**Published:** 2017-12-05

**Authors:** Hyeonyoung Ko, Jinyoung Shin, Jeong Eon Lee, Seok Jin Nam, Tuong Linh Nguyen, John Llewelyn Hopper, Yun-Mi Song

**Affiliations:** 1 Department of Family Medicine, Samsung Medical Center, Sungkyunkwan University School of Medicine, 81 Irwon-ro, Gangnam-gu, Seoul, 135-710 South Korea; 2 Department of Surgery, Samsung Medical Center, Sungkyunkwan University School of Medicine, Seoul, South Korea; 3 Melbourne School of Population and Global Health, Centre for Epidemiology and Biostatistics, University of Melbourne, Carlton, VIC Australia; 4 Department of Epidemiology, School of Public Health and Environment, Seoul National University, Seoul, South Korea; 5 Department of Family Medicine, Konkuk University Medical Center, Konkuk University School of Medicine, Seoul, South Korea

**Keywords:** Mammographic density, Ductal carcinoma in situ, Invasive ductal breast cancer

## Abstract

**Background:**

In spite of the increasing incidence of in situ breast cancer, the information about the risk factors of in situ breast cancer (DCIS) is scarce as compared to the information available for invasive ductal breast cancer (IDC), with inconsistent findings regarding the difference in risk factors between DCIS and IDC.

**Methods:**

We enrolled 472 women with IDC and 90 women with DCIS and 1088 controls matching for age and menopausal status. Information on risk factors was collected through self-administered questionnaire. Percent mammographic dense area (PDA), absolute mammographic dense area (ADA), and nondense area were assessed using a computer-assisted thresholding technique. Odds ratio (OR) and 95% confidence intervals (CI) were estimated by conditional logistic regression model with adjustment for covariates.

**Results:**

Later age at menarche and regular physical exercise were associated with decreased risk of IDC, whereas alcohol consumption, previous benign breast disease, and family history of breast cancer were associated with increased risk of IDC. For DCIS, previous benign breast disease and alcohol consumption were associated with the increased risk, and regular physical exercise was associated with decreased risk. Increase of ADA by 1-quartile level and PDA increase by 10% were associated with 1.10 (95% CI: 1.01, 1.21) and 1.10 (95% CI: 1.01, 1.19) times greater risk of IDC, respectively. The increase of ADA by 1-quartile level and PDA increase by 10% were associated with 1.17 (95% CI: 0.91, 1.50) times and 1.11 (95% CI:0.90,1.37) times greater risk of DCIS, respectively, but the associations were not statistically significant. There was no significant difference in the association with risk factors and mammographic density measures between IDC and DCIS (*P* > 0.1).

**Conclusions:**

Differential associations of DCIS with mammographic density and risk factors as compared with the associations of IDC were not evident. This finding suggests that IDC and DCIS develop through the shared causal pathways.

**Electronic supplementary material:**

The online version of this article (10.1186/s12885-017-3841-0) contains supplementary material, which is available to authorized users.

## Background

Over the past several decades, the incidence rate of in situ breast cancer has increased worldwide, probably due to the widespread use of mammograms for breast cancer screening [1–3].

Ductal carcinoma in situ (DCIS), the most common type of in situ breast cancer, is the proliferation of presumably malignant epithelial cells confined to the mammary ducts and lobules without evident stromal invasion through the basement membrane [4]. DCIS is considered as a precursor lesion of invasive ductal cancer (IDC) in the middle of progressive changes in nuclear features from normal breast tissue to invasive breast cancer [5]. Approximately four-fold higher risk of IDC was reported in women diagnosed with DCIS [6]. Long-term studies on women with DCIS treated by diagnostic biopsy alone revealed that not all but substantially large proportion of the women were diagnosed with IDC over the course of follow up [7]. Expression of biological markers such as estrogen receptor (ER), progesterone receptor (PgR), and human epidermal growth factor receptor-2 (HER2) was found to be similar between in situ component and invasive component in breast samples with both DCIS and IDC [8, 9]. In addition, the same tumor suppressor gene in chromosome 11 was mutated or missing in both invasive and in situ breast cancer [10], and a study that compared 12 susceptibility loci found no strong evidence of presence of a different association between DCIS and IDC [11].

However, findings regarding the difference in risk factors between DCIS and IDC were inconsistent, and the information about the risk factors of DCIS was less available than for IDC, especially for Asian women. Some studies reported similar associations with risk factors such as family history of breast cancer, previous breast biopsy, or parity between DCIS and IDC [11–14], whereas other studies reported differential association [15, 16]. Mammographic density (MD) reflects the relative amount of fat, connective tissue, and epithelial tissue in breast. Studies have consistently reported MD as a significant strong risk factor for breast cancer independent of other breast cancer risk factors, for western as well as for Asian women population [17, 18]. However, it is also controversial whether MD differentially affects the risk of developing breast cancer between DCIS and IDC. Yaghjyan L et al. [19] found that in situ breast cancer had a stronger association with MD measured by percent breast density than invasive breast cancer, whereas other reported no differential association with respect to MD between DCIS and IDC [20, 21].

We therefore conducted a case-control study in Korean women to evaluate the associations of breast cancer with risk factors including MD, separately for DCIS and IDC. Considering that most of the previous studies have been conducted on Western population and information on the risk factors of breast cancer by invasiveness for Asian women population was scarce, it is hypothesized that the findings from this study on the extent to which DCIS and IDC share the risk factors may provide awareness regarding the natural history of breast cancer in Asian women.

## Methods

### Study design and study subjects

We included a total of 562 breast cancer patients (472 IDC and 90 DCIS), who received curative surgery at Samsung Medical Center between February 2006 and August 2013 and had available data for MD and pathologic status. Of the 562 cases, 186 cases were recruited retrospectively from the Health Promotion Center of the Samsung Medical Center, while 376 cases were prospectively recruited from the department of surgery. Controls were randomly selected from 6863 women who had repeatedly (at least three times) received a periodic health checkup at the Health Promotion Center in the Samsung Medical Center and had no evidence of malignant breast disease for at least 1 year after the time at which mammogram for the present study was taken. We selected two controls for each breast cancer case through individual matching for menopausal status and age (within one year) at which mammogram was taken, except for 36 cases for whom only one control could be selected because of the limited control pool within matching strata. Thus, 1088 controls (912 for IDC cases and 176 for DCIS cases) were included in the final analysis. This study was approved by the Institutional Review Board of Samsung Medical Center (SMC 2011–06–052-022). The Board waived informed consent for the retrospectively recruited subjects, and all prospectively recruited subjects provided written informed consent.

### Mammographic density measurements

Mammograms were taken at the same institution using a full-field digital mammography system such as Senograph 2000D/DMR/DS (General Electric Company, Milwaukee, WI, USA) or Selenia (Hologic, Inc. Bedford, MA, USA). For breast cancer cases, MD of the breast contralateral to the breast involved in the cancer diagnosis was measured in the mammograms taken 1.0 (standard deviation: 2.1) months prior to the diagnosis. For controls, MD of the right breast was measured. Single observer who was blinded to all identifying information completed the measurement of total breast area (cm^2^) and area of mammographically dense tissue (ADA, cm^2^) directly from the cranio-caudal view using the computer-assisted thresholding technique (Cumulus: Imaging Research Program, Sunnybrook Health Sciences Centre, University of Toronto, Toronto, Canada). Subsequently, we calculated nondense area (cm^2^) of breast by subtracting ADA from total breast area and percentage dense area (PDA) as ADA divided by total breast area. MD measurement by Cumulus was reported to be highly reproducible [18]. Estimates of intraclass correlation coefficients for repeatedly measured MD were 0.99 for total breast area and 0.98 for ADA [19]. We categorized total breast area, nondense area, and ADA into four levels based on the quartile distribution of mammographic measures in the control group. PDA was categorized into five levels by 10% interval.

### Other measurements

We obtained information about pathological examinations and hormone receptor status by reviewing electronic medical records of the breast cancer cases. Expression of ER, PgR, and HER2 was assessed by immunohistochemistry staining kits: ER by 6F11 (Novocastra Laboratories, Newcastle upon Tyne, UK), PgR by IA6 (Novocastra Laboratories, Newcastle upon Tyne, UK), and HER2 by CB11 (Novocastra Laboratories, Newcastle upon Tyne, UK). ER and PgR positivity was defined as an Allred score of 3 to 8. Allred scoring semi-quantitatively measures the proportion of positive cells on 0 to 5 scales and staining intensity on a 0 to 3 scale. Positivity for HER2 overexpression was defined as a score of 3+ (strong, complete membrane immunoreactivity in >10% of tumor cells) on immunohistochemistry or as a gene amplification ratio ≥ 2.0 by fluorescence in situ hybridization using PathVysion HER2 DNA Probe kits (Abbott Molecular Inc., Des Plaines, IL, USA).

Family history of breast cancer among first-degree relatives (mother, daughter, or sister), previous benign breast disease; menstrual and reproductive history (age at menarche, menopausal status, use of estrogen replacement therapy, and number of live birth); and health-related behaviors (smoking, alcohol consumption, and physical activity) were collected using a self-administered questionnaire. All control subjects and retrospectively recruited 186 cases completed the questionnaire on the same day when they received a mammogram, and prospectively recruited cases completed the questionnaire when they were admitted to the hospital for surgical treatment. We defined a woman postmenopausal if she had no menstrual period for at least one year, has ever received hormone replacement therapy, or aged over 55 years. Study participants were divided into two or three categories for each of the following variables: alcohol consumption (ever, never), smoking (ever, never), frequency of regular physical exercise (≥ 1/week, < 1/ week), and use of hormone replacement therapy (ever, never). Body mass index (BMI, kg/m^2^) was calculated using measured height (cm) and weight (kg).

### Statistical analysis

For some variables with missing data (i.e., age at menarche), we imputed data using the average value for the variable among controls in the same age group. We compared demographic and clinical characteristics between cases and controls by paired t test and Cochran-Mantel-Haenszel chi-square test. We also compared demographic and clinical characteristics between IDC and DCIS cases by t test and chi-square test.

We estimated odds ratio (OR) with 95% confidence intervals (95% CI) for DCIS and IDC associated with MD and clinical risk factors by fitting a conditional logistic regression model for matched case-control study data. For estimating the association between MD and breast cancer, we adjusted covariates including age, menopausal status, height, BMI, age at menarche, number of live birth, smoking status, alcohol consumption, regular physical exercise, family history of breast cancer, previous benign breast disease, and use of estrogen replacement therapy. Furthermore, to reduce he probable confounding by the different recruitment method, we adjusted the recruitment method in addition.

We evaluated whether the association of breast cancer and clinical risk factors with MD differs between IDC and DCIS by adding interaction terms (invasiveness x each variable) to the analytic model.

In addition, we did stratified analysis according to the method of case recruitment and checked whether there is influence of recruitment method by examining interactions between the recruitment center and the variables on the breast cancer risk, separately for DCIS and IDC. We also did stratified analysis to examine whether the association of BMI with DCIS and IDC differed according to the menopausal status, and checked interaction between menopausal status and BMI, separately for DCIS and IDC.

All statistical analyses used the SAS statistical package (SAS Institute, Cary, NC, USA) with the level of statistical significance set as *P* = 0.05.

## Results

DCIS cases occupied 16.0% (90 cases) of all the breast cancer cases. Clinical and lifestyle characteristics and mammographic measures were compared between cases and controls, and between IDC and DCIS (Table 1). There was significant difference in BMI, age at menarche, number of live birth, use of estrogen replacement, alcohol consumption, smoking, physical exercise, previous benign breast disease, family history of breast cancer, and all MD measures between breast cancer cases and controls. Compared to IDC, DCIS cases had lower mean BMI and were less likely to be involved in frequent (≥3/week) regular physical exercise. Although IDC cases had greater total and nondense breast area, ADA and PDA did not differ between IDC and DCIS cases. There was no difference in ER and PgR positive status between IDC and DCIS cases, whereas DCIS cases were more likely to be HER2 positive than IDC cases were.

**Table 1:** Comparisons of demographic and clinical characteristics between cases and controls and between invasive ductal carcinoma and ductal carcinoma in situ

Variables	All cases (*n* = 562)	Invasive Ductal carcinoma (*n* = 472)	Ductal carcinoma in situ (*n* = 90)	Control (*n* = 1088)	P_difference*_	P_difference_†
Age at mammogram, years, mean (SD)	48.7(7.5)	48.9(7.6)	48.1(7.1)	49.3(7.1)	<0.001	0.358
Premenopausal, N (%)	389 (69.2)	323 (68.4)	66 (73.3)	742 (65.6)	–	0.356
Body mass index, kg/m^2^, mean (SD)	22.9(2.9)	23.1(3.0)	21.8(2.4)	22.5(2.8)	0.002	<0.001
Age at menarche, years, mean (SD)	14.7(1.7)	14.6(1.7)	14.8(1.7)	14.9(1.6)	0.003	0.357
Number of live birth, mean (SD)	1.9(0.9)	1.9(0.9)	1.8(1.0)	2.0(1.0)	0.001	0.714
Ever-use of estrogen replacement, N (%)	40 (7.1)	36 (7.6)	4 (4.4)	107 (9.8)	0.066	0.489
Ever alcohol consumption, N (%)	271 (48.2)	223 (47.3)	48 (53.3)	413 (38.0)	<0.001	0.290
Ever smoking, N (%)	46 (8.2)	40 (8.5)	6 (6.7)	54 (5.0)	0.009	0.566
Doing physical exercise ≥3/week, N (%)	185 (32.9)	164 (34.8)	21 (23.3)	280 (25.8)	0.001	0.035
Doing physical exercise, ≥ 1/week, N (%)	299(53.2)	248(52.5)	51(56.7)	902(82.9)	<0.001	0.472
Previous benign breast disease, N (%)	155 (27.6)	127 (26.9)	28 (31.1)	71 (6.5)	<0.001	0.413
Breast cancer among first degree relatives, N (%)	56 (10.0)	49 (10.4)	7 (7.8)	42 (3.9)	<0.001	0.450
Tumor marker status, N (%)						
Estrogen receptor (+)	444 (79.1)	373 (79.0)	71 (79.8)			0.873
Progesterone receptor (+)	401 (71.5)	334 (70.8)	67 (75.3)			0.387
Human epidermal growth factor receptor-2 (+)	134 (25.2)	103 (23.0)	31 (36.5)			0.009
Mammographic density measures						
Total breast area, cm^2^, mean (SD)	101.9(37.2)	103.9(38.0)	91.3(31.1)	94.9(33.6)	<0.001	0.001
Dense area, cm^2^, mean (SD)	20.4(14.5)	20.7(14.8)	19.1(13.0)	17.0(12.6)	<0.001	0.355
Nondense area, cm^2^, mean (SD)	81.5(36.3)	83.2(36.6)	72.1(33.2)	78.0(33.5)	0.033	0.008
Percent density, %, mean (SD)	21.4(13.5)	21.1(13.2)	22.8(14.9)	18.8(12.2)	<0.001	0.287

*SD* standard deviation, *N* number

*P value for the difference between cases and matched controls was assessed by paired t test for continuous variables or Cochran-Mantel-Haenszel chi-square test for categorical variables

†*P* value for the difference between the cases with invasive ductal carcinoma and the cases with ductal carcinoma in situ was assessed by student t test for continuous variables or Chi-square test for categorical variables

Table 2 shows the associations of DCIS and IDC with clinical characteristics after adjusting for other variables as compared to the matched controls. Later age at menarche (OR (95%CI): 0.94(0.88, 0.99)) and regular physical exercise for ≥1/week (OR (95%CI) were 0.45(0.37, 0.54) were associated with decreased risk of IDC, whereas alcohol consumption (OR (95%CI): 1.19(0.99, 1.44)), previous benign breast disease (OR (95%CI): 2.31 (1.86,2.86)), and history of breast cancer among first degree relatives (OR (95% CI):1.43(1.05, 1.95)) were associated with increased risk of IDC. For DCIS, alcohol consumption (OR (95% CI): 1.81 (1.14, 2.89)) and previous history of benign breast disease (OR (95%CI): 2.04 (1.23, 3.39)) showed a significantly increased risk. Regular physical exercise for ≥1/week (OR (95%CI): 0.52(0.31, 0.87)) was associated with decreased risk of DCIS. When we examined that the associations between candidate risk factors and breast cancer were modified by pathologic type of invasiveness, there was no significant difference in the association between IDC and DCIS (*P* > 0.1).

**Table 2:** Multivariable adjusted associations of clinical and reproductive characteristics with invasive ductal carcinoma and ductal carcinoma in situ

	Invasive ductal cancer				Ductal carcinoma in situ				*P* ^‡^ for interaction
	Measurement*		Association		Measurement*		Association		
Variables	Cases (*n* = 472)	Controls (*n* = 912)	OR (95% CI)^†^	*P* value	Cases (*n* = 90)	Controls (*n* = 176)	OR (95% CI)^†^	*P* value	
Body mass index, increase by 1 kg/m^2^	23.1 (3.0)	22.5 (2.8)	1.01 (0.98,1.04)	0.581	21.8 (2.4)	22.1 (2.8)	0.94 (0.86,1.03)	0.210	0.259
Age at menarche, increase by 1- year	14.6 (1.7)	14.9 (1.6)	0.94 (0.88,0.99)	0.030	14.8 (1.7)	14.8 (1.5)	1.08 (0.92,1.26)	0.370	0.320
Number of live birth, increase by 1-person	1.9 (0.9)	2.1 (1.0)	0.94 (0.85,1.03)	0.180	1.8 (1.0)	2.0 (1.0)	0.89 (0.71,1.11)	0.304	0.781
Ever-use of estrogen replacement	36 (7.6)	96 (10.5)	0.88 (0.59,1.30)	0.515	4 (4.4)	11 (6.3)	1.04 (0.31,3.41)	0.955	0.901
Ever alcohol consumption	223(47.3)	349(38.3)	1.19(0.99,1.44)	0.068	48(53.3)	64(36.4)	1.81(1.14,2.89)	0.013	0.105
Ever smoking	40(8.5)	43(4.7)	1.16(0.83,1.63)	0.381	6(6.7)	11(6.3)	0.96(0.37,2.53)	0.934	0.910
Regular physical exercise(≥1/week)	248(52.5)	754(82.8)	0.45(0.37,0.54)	<0.001	51(56.7)	148(84.1)	0.52(0.31,0.87)	0.013	0.434
Previous benign breast disease	49 (10.4)	39 (4.3)	2.31 (1.86,2.86)	<0.001	28 (31.1)	16 (9.1)	2.04 (1.23,3.39)	0.006	0.484
Breast cancer among first degree relatives	127 (26.9)	55 (6.0)	1.43 (1.05,1.95)	0.025	7 (7.8)	3 (1.7)	2.14 (0.84,5.44)	0.109	0.701

*Presented by mean (standard deviation) for continuous variables or number (%) for categorical variables

†Odd ratio (OR) and 95% confidence intervals (CI) were estimated by conditional logistic regression analysis after adjusting for age, menopausal status, height, body mass index, age at menarche, number of children, ever smoking status, alcohol consumption, regular physical exercise, family history of breast cancer among first degree relatives, past history of benign breast disease, use of estrogen replacement, and the method of recruiting subjects

‡Estimated by putting interaction term (each variable X invasiveness) in the conditional logistic regression model with adjustment for covariates

We checked the influence by the method of case recruitment (Additional file 1: Table S1). A significant interaction by the method of case recruitment was found on the association of IDC with ever-use of estrogen replacement and regular physical exercise, with different direction of association. Although there was a significant interaction between recruitment method and the history of previous benign disease on the risk of DCIS, the direction of the association was same with much higher OR in prospectively recruited subjects than retrospectively recruited subjects.

Table 3 shows the association of IDC and DCIS with each MD measure after adjusting for covariates. Total breast area and nondense area were not associated with the risk of both IDC and DCIS. ADA and PDA were positively associated with the IDC. Increase in ADA by 1-quartile level was associated with 1.10 (95% CI: 1.01, 1.21) times greater risk of IDC and 1.17 (95% CI: 0.91, 1.50) times greater risk of DCIS. Increase in PDA by 10% was associated with 1.10 (95% CI: 1.01, 1.19) times greater risk of IDC and 1.11 (95% CI: 0.90, 1.37) times greater risk of DCIS. Although the associations between DCIS and ADA, PDA were not statistically significant, there was no difference in the association with MD between IDC and DCIS: the P for interactions by invasiveness of breast cancer was 0.426 and 0.666 for the association of breast cancer with ADA and PDA, respectively.

**Table 3:** Multivariable adjusted association of mammographic density measures with invasive ductal carcinoma and ductal carcinoma in situ

	Invasive ductal cancer			Ductal carcinoma in situ		
	Number, Cases/controls	OR (95% CI)*	*P* value	Number, Cases/controls	OR (95% CI)*	*P* value
Total area (cm^2^) ^†^						
Q1 (< 71.2985)	95/220	1		23/52	1	
Q2 (71.2985–89.9320)	94/225	0.99(0.74,1.33)	0.957	23/47	1.42(0.75,2.72)	0.285
Q3 (89.9321–111.8983)	121/235	0.98(0.73,1.31)	0.981	28/37	1.93(0.96,3.90)	0.067
Q4 (111.8984 ≤)	162/232	1.17(0.86,1.58)	0.319	16/40	1.79(0.78,4.11)	0.171
1-quartile increase	472/912	1.05(0.95,1.16)	0.323	90/176	1.24(0.96,1.61)	0.095
P for Interaction^§^ = 0.998						
Absolute dense area (cm^2^) ^†^						
Q1 (< 8.09656)	82/230	1		19/42	1	
Q2 (8.09656–14.46536)	107/217	1.27(0.94,1.72)	0.116	14/55	0.59(0.27,1.28)	0.184
Q3 (14.46537–22.46433)	113/225	1.23(0.91,1.68)	0.176	32/47	1.11(0.54,2.27)	0.780
Q4 (22.46434 ≤)	170/240	1.41(1.05,1.90)	0.022	25/32	1.35(0.64,2.83)	0.434
1-quartile increase	472/912	1.10(1.01,1.21)	0.039	90/176	1.17(0.91,1.50)	0.218
P for Interaction^§^ = 0.426						
Non-dense area (cm^2^)^†^						
Q1 (< 54.8846)	109/219	1		31/53	1	
Q2 (54.8846–70.4895)	92/231	0.87(0.65,1.16)	0.330	20/41	1.41(0.73,2.72)	0.306
Q3 (70.4896–93.2822)	120/236	0.93(0.70,1.24)	0.618	22/36	1.74(0.84,3.61)	0.134
Q4 (93.2823 ≤)	151/226	1.03(0.75,1.41)	0.849	17/46	1.48(0.62,3.49)	0.376
1-quartile increase	472/912	1.02(0.92,1.13)	0.747	90/176	1.17(0.90,1.53)	0.251
P for Interaction^§^ = 0.693						
Percentage dense area ^‡^						
< 10%	98/248	1		21/48	1	
10–19%	151/285	1.29(0.97,1.70)	0.076	17/60	0.68(0.33,1.43)	0.310
20–29%	116/219	1.37(1.01,1.85)	0.044	32/42	1.19(0.58,2.45)	0.641
30–39%	62/98	1.36(0.95,1.95)	0.095	7/24	0.55(0.19,1.58)	0.267
≥ 40%	45/62	1.54(1.02,2.31)	0.039	13/2	1.90(0.72,5.06)	0.197
10% increase	472/912	1.10(1.01,1.19)	0.029	90/176	1.11(0.90,1.37)	0.348
P for Interaction^§^ = 0.666						

*Odd ratio (OR) and 95% confidence intervals (CI) were estimated by conditional logistic regression analysis after adjusting for age, menopausal status, height, body mass index, age at menarche, number of children, ever smoking status, alcohol consumption, regular physical exercise, family history of breast cancer among first degree relatives, past history of benign breast disease, use of estrogen replacement, and the method of recruiting subjects

†Quartiles (Q) were determined based on the distribution of mammographic measures of control group. Q1 is the lowest quartile level and Q4 is the highest quartile level

‡Calculated as the dense area divided by total breast area

§Estimated by putting interaction term (unit of increase in each mammographic density measure X invasiveness) in the conditional logistic regression model after adjusting for covariates

## Discussion

In the present case-control study on Korean women, the direction and the size of estimates for the association of DCIS with reproductive factors and MD were similar to those of IDC, and no significant heterogeneity in the association between DCIS and IDC was found.

Mammographic density is a well-established strong risk factor for invasive breast cancer [17]. Epidemiologic studies have revealed significant association between breast in situ cancer and MD [21–23]. Interestingly, some study findings suggested the possibility of existence of stronger association between MD and DCIS than that between MD and IDC. A case study found that most of the DCIS lesions (21 of 22) occurred from areas of dense tissue [24]. In a study of Canadian cohort, the relative risk for detecting breast atypia or DCIS in biopsy specimens from women with more than 75% density was estimated to be 9.7 times higher when compared with that from women showing no mammographic density [25]. A possible explanation for the probably stronger association of MD with DCIS than that with IDC was that radiographic appearance of in situ cancer might result in higher sensitivity of screening mammography for detection of DCIS as compared with IDC detection [26]. In accordance with this suggestion, in a nested case-control study, the OR (6.58, 95% CI = 3.47, 12.48) for in situ breast cancer associated with the highest category of PDA (≥50%) as compared with the lowest PDA (<10%) was significantly higher than the OR (3.00, 95% CI: 2.13, 4.23) for invasive breast cancer (P for heterogeneity <0.01) [19]. However, other studies showed that the association of MD did not differ between in situ cancer and invasive cancer. A case only study by Ghosh et al. [27], revealed no difference in the ADA and PDA between IDC, DCIS, invasive lobular cancer, and lobular carcinoma in situ after adjusting for covariates. In a nested case-control study within the multiethnic cohort, for the highest category of PDA (≥50%) and ADA (≥45cm^2^) as compared with the lowest (<10%, <15cm^2^), the ORs were 3.58 (95% CI: 2.26, 5.66) and 2.92 (95% CI: 2.01, 4.25) for IDC, and 2.86 (95% CI: 1.38, 5.94) and 2.59 (95% CI: 1.39, 4.82) for DCIS [20] without statistically significant difference between IDC and DCIS [20]. A large study including 3414 cases and 7199 controls also found 2.21(95% CI: 1.92, 2.55) and 1.87 (95% CI: 1.42, 2.48) times higher risk of IDC and DCIS, respectively for high (>51%) versus average (11–25%) density group, also without significant heterogeneity [28]. A British case-control study reported that the OR of IDC (1.3) associated with denser breast as compared with less dense breast was similar to the OR of DCIS (1.3) [23]. In our study, although the association between MD and DCIS did not reach statistical significance, the risk estimates for the association of DCIS with both ADA and PDA were almost similar with those for IDC, and they did not significantly differ from the risk estimate for IDC, as found in the British study [23].

Interestingly, the estimates (OR (95% CI) for the association between MD and breast cancer in our study tend to be weaker than the magnitude of association observed in the above mentioned studies in Western population: the risk associated PDA ≥ 40% was 1.54 (1.02, 2.31) for IDC and 1.90 (0.72, 5.06) for DCIS in our study. Different strength of association between MD and breast cancer has been frequently reported across different ethnic groups [29, 30], and the association observed in Asian women tended to be weaker than the association in women from Western populations [31, 32]. In a previous meta-analysis, the relative risk ratio of developing breast cancer for women in Wolfe’s most-dense category (DY) compared with those in the least-dense category (N1) was 3.98 (95% CI: 2.53, 6.27) from an incidence study, and 2.42 (95% CI: 1.98, 2.97) from a prevalence study [32]. In comparison, from a Japanese case-control study, the relative risk was 2.20 (95% CI: 1.02, 4.77) for DY group compared with N1 groups [31]. However, because of the lack of Asian studies on MD and different pathologic type of breast cancer, we could not directly compare the difference in the strengths of association of IDC and DCIS between Western and Asian population.

The association between age at menarche and in situ cancer was controversial with either null [12, 13, 16] or inverse association [33]. In our study, age at menarche had an inverse association with IDC, but not with DCIS. However, no heterogeneity regarding the association with the age at menarche existed between IDC and DCIS. We assume that this conflicting finding might have been caused by the inadequate sample size of DCIS in our study. However, given that some studies with large enough sample size have reported no association [12, 13], the association between age at menarche and DCIS in Asian population needs further evaluation in a study with large enough sample size.

In the present study, we found no significant association of BMI with IDC as well as DCIS. In studies that did not differentiate premenopausal and postmenopausal breast cancer, no association between BMI and in situ cancer and a positive association between BMI and invasive cancer have been reported. [13, 16] Most of the previous studies have reported presence of significant inverse association between BMI and DCIS in premenopausal women [12, 33–36]. The relation between BMI and postmenopausal DCIS has not been clarified yet with conflicting findings with null [11, 13, 16, 21, 34], positive [33], or inverse [37] association. A study on premenopausal women reported a stronger inverse association for in situ cancer than the association for invasive cancer (<45). [12] We suppose the findings in studies of mixed premenopausal and postmenopausal women could have been influenced by the proportion of postmenopausal women among IDC and DCIS cases. Although interaction by menopausal status on the association of both IDC and DCIS with BMI was not evident in our study (Additional file 1: Table S2), the association of obesity with breast cancer needs to be further evaluated with consideration of pathologic type and menopausal status.

It has been suggested that the risk factors operating early in life such as family history might be involved in the initial stages of carcinogenesis, resulting in in situ cancer, and other factors needed to continue promoting the tumor to invasive cancer [13]. A study that found stronger association of a family history of breast cancer with DCIS than with invasive cancer, especially in younger women than in older women suggested greater genetic influence on DCIS [34]. On the other hand, the increased risk associated with the breast cancer of at least one first degree relative has been consistently similar between IDC and DCIS in many studies [21, 34, 37], suggesting an inherited predisposition to both types of breast cancer. Our study also confirmed family history of breast cancer is an important risk factor of IDC as well as DCIS. Although the association was borderline significant for DCIS, the estimate for DCIS (OR = 2.14) was greater in strength than that for IDC (OR = 1.43). Given that a woman with a family history of breast cancer is more likely to volunteer to health check-up, the risk of breast cancer associated with family history of breast cancer might have been underestimated in our study. Thus, the positive association of family history with DCIS and IDC in our study seems to provide strong evidence supporting the role of genetic effect on breast cancer. Although differential association with DICS versus IDC has been found for some breast cancer predisposition loci, most (76%) of breast cancer predisposition loci previously reported for IDC were associated with DCIS in the same direction in several studies [11, 38, 39], which support strong shared genetic susceptibility of DCIS and IDC.

The risks of IDC and DCIS have been consistently found to increase in women with a history of benign breast disease [12, 16, 33]. Our study also found that benign breast disease is associated with increased risk of breast IDC as well as DCIS and the risk estimates for DCIS (OR: 2.04) were similar to that for IDC (OR: 2.31).

Regular physical activity has been proposed as an independent protective factor of breast cancer [40, 41], which we confirmed in our study. It has been scarcely evaluated whether the association with physical activity differs between IDC and DCIS. In a case-control study by Trentham-Dietz et al. [16], increasing frequency of physical activity in early adulthood was inversely associated with the risk of invasive cancer (OR(95% CI) per frequency/week: 0.96(0.93,0.99)), and it did not significantly differ from the association between in situ cancer and physical activity (OR(95% CI): 0.99(0.92,1.07)). We also found that regular physical activity was inversely associated with the risk of IDC as well as DCIS and the estimates were not different each other. Studies have consistently reported that alcohol consumption had a positive association with breast cancer [42, 43]. In the present study, we found that DCIS have a stronger association with alcohol consumption than IDC had, although the difference was not statistically different (*P* = 0.105). Trentham-Dietz et al. also reported that ORs (95% CI) for ≥183 g/week of alcohol intake were 2.34(1.32, 4.16) for DCIS and 1.76(1.37, 2.25) for IDC but without statistical difference between them [16], which is very consistent with the findings of our study.

The present study had some limitations. First, our study may have weakness with respect to representativeness that is commonly innate in a hospital-based case-control study. As controls were recruited from participants in a health checkup program, selection bias may exist. We tried to overcome this bias by considering a wide range of covariates including health behaviors such as physical exercise, alcohol consumption, and smoking habit. Second, we recruited cases in two ways (retrospectively or prospectively), and this might have incurred bias in the study findings due to the health behavior modification or recall bias. To reduce the probable confounding by the heterogeneous recruitment method, we adjusted for the recruitment method. However, the significant interaction by the recruitment method on the association of IDC with physical exercise and ever-use of estrogen replacement with different direction of the estimates for the association between the retrospectively (positive) recruited subjects and prospectively (inverse) recruited subjects suggests that careful interpretation is necessary, especially for the association of IDC and those two factors. Third, we could not consider the mode of breast cancer detection and could not examine the association of age at menopause, age at first birth, lactation, and oral pill with IDC and DCIS because of the lack of information. Fourth, because of the low proportion of DCIS among breast cancer, we may have included relatively small number of DCIS cases in this study and, thus study power could have been inadequate. Finally, given that ‘benign breast disease’ constitutes a heterogeneous group of breast lesions, evaluation of breast cancer risk associated with the benign breast disease could have been too vague to give useful clinical information.

On other hand, our study has some strength. First, the influence of age and menopausal status was strictly controlled through individual case-control matching and statistical adjustment. Second, a wide range of covariates was considered: BMI, age at menarche, number of children, use of estrogen replacement therapy, lifestyle factors, previous benign breast disease, and family history of breast cancer among first-degree relatives. Third, we measured MD quantitatively using a computer-assisted thresholding technique.

## Conclusions

In conclusion, differential associations of DCIS with mammographic density and risk factors as compared with the associations of IDC were not evident. This finding suggests that IDC and DCIS develop through the shared causal pathways.

## Additional file

Additional file 1: Table S1. Influence of the method of subjects recruitment on the association of the clinical, reproductive, and mammographic density characteristics with invasive ductal carcinoma and ductal carcinoma in situ. **Table S2** Association of body mass index with breast cancer according to menopausal status. (DOCX 23 kb)

## Abbreviations

ADA: absolute mammographic dense area
DCIS: ductal carcinoma in situ
ER: estrogen receptor
HER2: Cerb2 receptor
IDC: invasive ductal cancer
MD: mammographic density
PDA: percent mammographic dense area
PgR: progesterone receptor
