# Supplementary material for: Comparison of the association of mammographic density and clinical factors with ductal carcinoma in situ versus invasive ductal breast cancer in Korean women
Source: BMC Cancer. 2017 Dec 5;17:821. doi: 10.1186/s12885-017-3841-0 (PMC5718024; doi:10.1186/s12885-017-3841-0)
Supplement: Additional file 1: Table S1. — Influence of the method of subjects recruitment on the association of the clinical, reproductive, and mammographic density characteristics with invasive ductal carcinoma and ductal carcinoma in situ. Table S2 Association of body mass index with breast cancer according to menopausal status. (DOCX 23 kb) [file 12885_2017_3841_MOESM1_ESM.docx]

| Supplementary Table 1. Influence of the method of subjects recruitment on the association of the clinical, reproductive, and mammographic density characteristics with invasive ductal carcinoma and ductal carcinoma in situ. | | | | | | |
| --- | --- | --- | --- | --- | --- | --- |
|  | Invasive ductal cancer (N of cases=472) | | | Ductal carcinoma in situ (N of cases=90) | | |
|  | Retrospectively  Recruited  (N of cases=136) | Prospectively recruited  (N of cases=336) | P interaction^†^ | Retrospectively  Recruited  (N of cases=50) | Prospectively recruited  (N of cases=40) | P interaction^†^ |
|  | OR (95% CI)^*^ | OR (95% CI)^*^ |  | OR (95% CI)^*^ | OR (95% CI)^*^ |  |
| Body mass index, increase by 1kg/m^2^ | 1.02(0.95,1.09) | 1.00(0.97,1.04) | 0.661 | 0.93(0.82,1.06) | 1.06(0.88,1.27) | 0.322 |
| Age at menarche, increase by 1- year | 0.96(0.86,1.07) | 0.94(0.87,1.01) | 0.898 | 1.01(0.80,1.26) | 1.20(0.91,1.59) | 0.500 |
| Number of live birth, increase by 1-person | 0.95(0.79,1.13) | 0.89(0.79,1.01) | 0.232 | 0.96(0.72,1.29) | 0.76(0.43,1.32) | 0.436 |
| Ever-use of estrogen replacement | 1.60(0.88,2.92) | 0.51(0.27,0.96) | 0.007 | 1.23(0.33,4.49) | - | - |
| Ever alcohol consumption | 1.05(0.72,1.51) | 1.18(0.94,1.49) | 0.848 | 1.80(0.94,3.46) | 1.76(0.76,4.04) | 0.867 |
| Ever smoking | 1.39(0.73,2.63) | 1.11(0.73,1.68) | 0.906 | 0.68(0.19,2.43) | 4.01(0.65,24.9) | 0.184 |
| Regular physical exercise (≥ 1/week) | 1.05(0.69,1.59) | 0.33(0.26,0.41) | <0.001 | 0.50(0.24,1.04) | 0.31(0.09,1.04) | 0.266 |
| Previous benign breast disease | 1.71(1.03,2.84) | 2.47(1.93,3.17) | 0.373 | 1.27(0.52,3.08) | 4.30(1.74,10.64) | 0.037 |
| Breast cancer among first degree relatives | 1.90(1.02,3.51) | 1.30(0.89,1.89) | 0.441 | 1.81(0.55,5.95) | 3.12(0.35,28.01) | 0.568 |
| Mammographic density measures |  |  |  |  |  |  |
| Total area, 1-quartile increase | 0.98(0.82,1.19) | 1.08(0.96,1.22) | 0.278 | 1.31(0.93,1.05) | 1.02(0.60,1.73) | 0.499 |
| Absolute dense area, 1-quartile increase | 0.98(0.81,1.18) | 1.12(1.00,1.25) | 0.245 | 1.40(0.98,2.00) | 1.04(0.65,1.67) | 0.547 |
| Non-dense area, 1-quartile increase | 0.97(0.79,1.19) | 1.04(0.92,1.18) | 0.311 | 1.12(0.80,1.57) | 1.04(0.55,1.97) | 0.661 |
| Percentage dense area, 10% increase | 1.13(0.93,1.38) | 1.07(0.97,1.17) | 0.358 | 1.34(0.91,1.96) | 1.04(0.74,1.45) | 0.572 |
| N: number  ^*^ Odd ratio (OR) and 95% confidence intervals (CI) were estimated by conditional logistic regression analysis, after adjusting for age, menopausal status, height, body mass index, age at menarche, number of children, ever smoking status, alcohol consumption, regular physical exercise, family history of breast cancer among first degree relatives, past history of benign breast disease, and use of estrogen replacement.  ^†^Interactions were estimated by putting interaction term (each variable x recruitment method) in the conditional logistic regression model. | | | | | | |

| Supplementary Table 2. Association of body mass index with breast cancer according to menopausal status. | | | | |
| --- | --- | --- | --- | --- |
|  | | Postmenopausal | Premenopausal | P for interaction^†^ |
| Overall breast cancer | N of case/controls | 173/346 | 389/742 |  |
|  | OR (95% CI)^*^ | 1.00(0.9,1.06) | 1.00(0.97,1.04) | 0.7324 |
| Invasive ductal cancer | N of case/controls | 149/298 | 323/614 |  |
|  | OR (95% CI)^*^ | 1.01(0.95,1.08) | 1.00(0.97,1.04) | 0.5877 |
| Ductal carcinoma in situ | N of case/controls | 24/48 | 66/128 |  |
|  | OR (95% CI)^*^ | 0.91(0.73,1.15) | 0.97(0.87,1.09) | 0.5807 |
| N: number  ^*^ Odd ratio (OR) and 95% confidence intervals (CI) were estimated by conditional logistic regression analysis, after adjusting for age, height, age at menarche, number of children, ever smoking status, alcohol consumption, regular physical exercise, family history of breast cancer among first degree relatives, past history of benign breast disease, use of estrogen replacement, the method of recruiting subjects.  ^†^Interactions were estimated by putting interaction term (body mass index X menopausal status) in the conditional logistic regression model. | | | | |
